# Mesothelin-Associated Anti-Senescence Through P53 in Pancreatic Ductal Adenocarcinoma

**DOI:** 10.3390/cancers17122058

**Published:** 2025-06-19

**Authors:** Dongliang Liu, Jianming Lu, Changyi Chen, Qizhi Yao

**Affiliations:** 1 Michael E. DeBakey Department of Surgery, Baylor College of Medicine, Houston, TX 77030, USA; donglial@bcm.edu (D.L.); jian-ming.lu@bcm.edu (J.L.); jchen@bcm.edu (C.C.); 2 Center for Translational Research on Inflammatory Diseases (CTRID), Michael E. DeBakey VA Medical Center, Houston, TX 77030, USA

**Keywords:** pancreatic ductal adenocarcinoma, mesothelin, cellular senescence, senescence-associated secretory phenotype, interleukin-8

## Abstract

**Objectives**: Mesothelin (MSLN) is overexpressed in pancreatic ductal adenocarcinoma (PDAC), promoting cell proliferation, migration, and inhibiting apoptosis. While its oncogenic properties have been documented, the role of MSLN in regulating cellular senescence—a tumor-suppressive mechanism—has remained unexplored. This study is the first to identify and characterize a novel mesothelin-associated anti-senescence (MAAS) effect in PDAC. **Methods**: A proteogenomic analysis of PDAC tissue samples from the Clinical Proteomic Tumor Analysis Consortium (CPTAC) was performed to evaluate MSLN-associated senescence pathways using WebGestalt. Human and murine PDAC cell lines with modified MSLN expression were analyzed for senescence phenotypes via SA-β-gal staining, Western blotting of key regulators (P53, P21^waf1^, and P16^ink4a^), γH2AX immunoblotting, and IL-8 quantification using ELISA. **Results**: The CPTAC analysis revealed an inverse correlation between MSLN expression and DNA damage/repair pathways. MSLN-deficient cells exhibited classic senescence features—growth arrest, an enlarged morphology, and elevated SA-β-gal activity. The expression of P53, P21^waf1^, and P16^ink4a^ was upregulated, alongside increased γH2AX levels, indicating the activation of the DNA damage response. IL-8 secretion was significantly higher in the MSLN knockdown cells and reduced in the MSLN-overexpressing cells, consistent with the modulation of the SASP. Notably, MSLN deficiency impaired cell viability without inducing overt cytotoxicity, supporting a shift toward senescence. **Conclusions**: Our findings uncover a previously unrecognized mechanism through which MSLN promotes tumor progression by suppressing senescence via P53-associated pathways. Targeting the MAAS pathway may offer a novel therapeutic strategy to restore tumor-suppressive senescence and enhance treatment efficacy in PDAC.

## Simple Summary

Pancreatic ductal adenocarcinoma (PDAC) is a deadly disease with limited treatment options. This study focuses on the protein mesothelin (MSLN), which is highly expressed in over 89% of clinical PDAC specimens and helps cancer cells grow and avoid death. This study aims to determine how MSLN prevents cancer cells from entering a state called senescence, where they stop growing. The results show that silencing *MSLN* gene expression in PDAC cells induced senescence phenotypes, including growth arrest and an increased expression of senescence markers (P53, P21^waf1^, and P16^ink4a^), along with higher IL-8 production. Mechanically, high DNA damage/DNA repair activities associated with low MSLN expression may activate the senescence pathway in pancreatic cancer cells. Our findings could lead to better treatment options for pancreatic cancer, potentially improving outcomes for patients.

## 1. Introduction

Pancreatic ductal adenocarcinoma (PDAC) is one of the most lethal cancers and is characterized by an insidious onset, a poor prognosis, and distal metastasis at diagnosis [1]. Due to its late diagnosis and resistance to chemotherapy and immunotherapy, PDAC remains one of the most intractable and devastating malignancies, with a median survival time of merely 6 months post-diagnosis and a 5-year survival rate of less than 7% [2]. Because of its increasing incidence and low cure rate, by 2030, PDAC is projected to exceed liver cancer and become the second leading cause of cancer death for both the male and female sexes in the United States [3]. Deciphering the molecular mechanisms of PDAC’s progression is necessary to design better treatment options.

Cell senescence is considered a growth arrest response to various cellular stresses, including DNA damage, oxidative stress, oncogene activation, etc. [4]. Senescence has been identified as a well-known tumor-suppressive mechanism because the inactivation of senescence effector programs leads to increased tumorigenesis in vivo, indicating that senescence represents an important barrier to the malignant progression of tumors [5]. Senescence-related proteins P53, P21, and P16 are frequently observed to be dysfunctional in many human cancers, which strongly correlates with a worse prognosis [6]. Oncogene-induced senescence (OIS) is crucial for protection against cancer development; it is an anti-proliferative cellular response to a series of oncogenic signaling due to activating mutations of oncogenes or the inactivation of tumor-suppressive genes [7]. The normal ductal epithelium transforming into PDAC undergoes acinar–ductal metaplasia (ADM) and develops into pancreatic intraepithelial neoplasia stages 1 to 3 (PanIN-1 to PanIN-3), with PanIN-3 generally considered to be ductal carcinoma in situ [8]. During the development of PDAC, senescence is reported to be observed only in the ADM and PanIN-1 periods and not in the PDAC stage [9,10]. In a KPC mouse model, pancreatic senescence was observed in PanIN lesions of the pancreas in 6~8-month-old mice, which expressed oncogenic Kras from their endogenous promoters; however, additional oncogenic events or cooperating genetic alterations are needed to break the balance between cellular senescence and proliferation to eventually promote tumor progression [9,10,11].

Mesothelin (MSLN) is a glycoprotein that is highly expressed in PDAC but not in normal pancreas tissues [12,13]. MSLN mediates cellular adhesion by binding to its receptor MUC16 [14,15]. Our previous studies revealed that MSLN acts as a malignant factor in PDAC by promoting cell proliferation and migration and contributing to tumor progression in different mouse models [16,17,18]. However, it is not clear at which point the expression of MSLN emerges during the development of PDAC, while altered MSLN expression has not been shown in the ADM and pancreatic intraepithelial neoplasia stages in immunohistochemical analyses [19]. Notably, MSLN is not expressed in normal pancreas and the ADM and PanIN-1 periods in which senescence can occur, while PDAC without senescence shows aberrantly high MSLN expression [8,9,10]. Although these findings suggest an inverse correlation between MSLN expression and cellular senescence, the specific role of MSLN in regulating senescence in cancer remains unclear.

Hence, the aim of this study was to investigate the potential relationship between MSLN and senescence in the development of PDAC and to obtain insights into the molecular mechanism of MSLN in regulating cellular senescence. This work is the first to demonstrate that MSLN actively suppresses senescence in PDAC cells, representing a novel function beyond its established roles in proliferation and metastasis. By defining a mesothelin-associated anti-senescence (MAAS) effect, our findings reveal a previously unrecognized mechanism of tumor progression and suggest that targeting MSLN could restore senescence, providing a new avenue for therapeutic intervention in PDAC.

## 2. Materials and Methods

### 2.1. Cells, Antibodies, and Cell Line Generation

Human PDAC cell lines AsPC1, CFPAC1, MIA Paca2, and Panc1 were purchased from the American Type Culture Collection (ATCC, Manassas, VA, USA) and maintained in Yao Lab. Human PDAC cell line Panc28 was obtained from MD Anderson Dr. Craig Logsdon’s lab. Murine PDAC Panc02 cell line was obtained as a gift from Dr. Sabry EL-Naggar, the Medical University of South Carolina [20]. If not specifically stated, all cells were cultured in 1× DMEM (10-017-CM, Corning, Corning, NY, USA ) or 1× RPMI-1640 (10-041-CV, Corning, Corning, NY, USA) medium supplemented with 10% *v*/*v* heat-inactivated Fetal Bovine Serum (35-011-CV, Corning, Corning, NY, USA), 100 units/mL penicillin, 100 μg/mL streptomycin, and 250 ng/mL amphotericin B (17-745E, Lonza, Basel, Switzerland). All cell lines used in the study were authenticated with the Short Tandem Repeat (STR) profiling technique by the Cytogenetics and Cell Authentication Core, MD Anderson Cancer Center, University of Texas.

Antibodies anti-MSLN (99966), anti-P53 (13684), anti-P21 (8242), anti-P16 (3033), and anti-H2A.X (7631S) were purchased from Cell Signaling Technology, Danvers, MA, USA; anti-γH2A.X. (AB2893) was purchased from Abcam, Cambridge, UK; and anti-GAPDH (G8795) was obtained from Sigma-Aldrich, St. Louis, MO, USA.

Genetically modified PDAC cell lines with MSLN knockdown, knockout, or overexpression were established as performed previously and maintained by Yao Lab [12,17]. Specifically, CRISPR/Cas9-mediated knockout of MSLN was performed using lentiviral delivery of sgRNA and Cas9. For human PDAC cell lines, lentiviral vectors with sgRNAs targeting MSLN (#HCP290262-LvSG03) and the control (#CCPCTR01-LvSG03) were purchased from GeneCopoeia, Inc., Rockville, MD, USA. Lentivirus was produced in HEK293T cells and used to infect target cells in the presence of polybrene (8 μg/mL). Infected cells were selected with puromycin (5 μg/mL) for 7 days. Following selection, cells were clonally expanded by limiting dilution to establish knockout clones. Gene editing efficiency was confirmed by Sanger sequencing and Tracking of Indels by Decomposition (TIDE) analysis, with >85% of reads showing insertions/deletions (indels) at the target locus across clones. For mouse PDAC cell lines, sgRNAs targeting a murine Msln vector (#MCP301309-LvSG03) and the control vector (#CCPCTR01-LvSG03) were purchased from GeneCopoeia, Inc., Rockville, MD, USA, and delivered via lentiviral transduction. Clonal expansion and validation were performed as described above. MSLN knockdown was achieved using shRNA constructs targeting MSLN (#HSH090262-LVRH1MP) from GeneCopoeia, Inc., Rockville, MD, USA. A scrambled vector (#CSHCTR001-LVRH1MP) was used as a control. Lentivirus was produced in HEK293T cells and used to infect target cells. Stable knockdown was selected using puromycin (5 μg/mL) for 7 days. Knockdown efficiency was confirmed using a Western blot. MSLN overexpression was performed by the lentiviral transduction of full-length human MSLN cDNA cloned into the pLenti6.3/V5™-TOPO™ vector (K531520, Thermo Fisher Scientific Inc., Waltham, MA, USA). Transduced cells were selected using blasticidin (5 μg/mL) for 7 days and validated by Western blot.

### 2.2. Pathway Enrichment Analysis

To investigate whether MSLN is associated with senescence-related pathways, we analyzed the RNA and protein expression profiles in PDAC patient tissues from the National Cancer Institute’s Clinical Proteomic Tumor Analysis Consortium (CPTAC) [21]. The trans-association correlations in PDAC between MSLN and all other top-rated protein coding genes at mRNA and protein levels were analyzed using LinkedOmicsKB (https://kb.linkedomics.org*/*, accessed on 6 March 2025) [22]. A gene set enrichment analysis (GSEA) was performed using the WEB-based GEne SeT AnaLysis Toolkit (WebGestalt, http://www.webgestalt.org/, accessed on 6 March 2025), and the significant genes or proteins from the trans-association results were used as an input list to conduct a pathway enrichment analysis using the KEGG pathway resource [23].

### 2.3. Senescence-Associated β-Galactosidase Assay (SA-β-Gal) and Cell Viability Assay

The colorimetric SA-β-Gal assay was conducted using the methods described by Fuhrmann-Stroissnigg et al. [24]. Briefly, MSLN gene knockout (MSLN-KO) or scramble PDAC cells were washed three times with cold PBS and then fixed with 2% formaldehyde and 0.2% glutaraldehyde in PBS for 10 min. Following fixation, cells were incubated in SA-β-Gal staining solution (1 mg/mL 5-bromo-4-chloro-3-indolyl-beta-d-galactopyranoside (X-gal), 1× citric acid/sodium phosphate buffer (pH 6.0), 5 mM potassium ferricyanide, 5 mM potassium ferrocyanide, 150 mM NaCl, and 2 mM MgCl_2_) at 37 °C for 16–18 h. The enzymatic reaction was stopped by washing the cells three times with ice-cold PBS. The cells were counterstained with Hoechst solution and analyzed with a fluorescence microscope.

Cell viability was assessed directly in 24-well plates using the 0.2% Trypan Blue exclusion assay. Briefly, the cells were gently washed with phosphate-buffered saline (PBS) to eliminate residual serum. Subsequently, 200 µL of 0.2% Trypan Blue solution was added to each well, and the mixture was incubated at room temperature for 5 min. After incubation, the excess dye was gently aspirated, and the cells were immediately visualized under an inverted light microscope. Viable cells appeared clear, while non-viable cells were stained blue. The numbers of stained (non-viable) and unstained (viable) cells were counted from representative fields in each well.

The quantification of SA-β-gal-positive cells or Trypan Blue-stained cells was conducted using ImageJ software (version 1.53t, National Institutes of Health, Bethesda, MD, USA) [25]. The brightfield images were converted to 8-bit grayscale and analyzed using the Color Deconvolution plugin to distinguish positively stained (blue) cells from the background. Positive cells were counted using the Cell Counter plugin or the Analyze Particles function after segmentation. The percentage of SA-β-gal-positive cells was calculated by dividing the number of blue-stained cells by the total number of cells per field. For the Trypan Blue exclusion assay, the percentage of viable cells was calculated by dividing the number of unstained (viable) cells by the total number of cells per field. A minimum of three randomly selected fields and three independent biological replicates were analyzed for each condition.

### 2.4. Western Blot Assay

Protein purification and a Western blot assay were carried out as previously described [12]. Cell pellets were collected and lysed using 1× RIPA Lysis Buffer (20-188, Millipore Sigma, Burlington, MA, USA) supplemented with 1× Protease and Phosphatase Inhibitor Cocktail (78440, Thermo Scientific, Waltham, MA, USA); following lysis, all cell lysates were centrifuged at 12,000 rpm for 15 min at 4 °C, and the protein concentration of supernatant was determined by using a PierceTM BCA Protein Assay Kit (23227, Thermo Scientific, Waltham, MA, USA). A Western blot assay was used to detect MSLN, while senescent markers and DNA damage response markers were determined with antibodies anti-MSLN (99966), anti-P53 (13684), anti-P21 (8242), anti-P16 (3033), anti-H2A.X (7631S) (Cell Signaling Technology, Danvers, MA, USA), anti-γH2A.X. (AB2893, Abcam, Cambridge, UK), and anti-GAPDH (G8795, Sigma-Aldrich, St. Louis, MO, USA) according to the manufacturer’s instructions. Immunoreactivity protein complexes were detected by using horseradish peroxidase (HRP)-conjugated anti-rabbit (7074) or anti-mouse IgG antibodies (7076) (Cell Signaling Technology, Danvers, MA, USA) and SuperSignal™ West Pico PLUS Chemiluminescent Substrate (34580, Thermo Scientific, Waltham, MA, USA). For blot quantification, the Gel Analysis tool in ImageJ was used [25]. Bands were selected using the rectangular selection tool, and intensity profiles were plotted to measure the area under the curve for each band. Band intensities were normalized to loading control, and the results were expressed as relative expression levels. Quantification was based on triplicate biological replicates.

### 2.5. Enzyme-Linked Immunosorbent Assay (ELISA)

The chemokine IL-8/CXCL-8, a very common senescence-associated secretory phenotype overexpressed by most senescent cells [26], was measured by using the LEGEND MAX™ Human IL-8 ELISA Kit (431507, BioLegend, San Diego, CA, USA) according to the manufacturer’s instructions. The optical density values were measured using a microplate reader at 450 and 570 nm, and values at 570 nm were subtracted from the absorbance at 450 nm for subsequent data analysis.

### 2.6. Statistical Analysis

All quantifications from the SA-β-gal staining and Trypan Blue staining assays reflect at least three samples with at least 100 events counted (typically in three different areas) each. Unless stated, the data are presented as arithmetic means ± standard deviation (s.d.), and statistical analyses were conducted based on unpaired two-sided *t*-tests. *p* < 0.05 was considered statistically significant. Data analyses were performed by using GraphPad Prism Version 8.4 [27].

## 3. Results

### 3.1. MSLN Negatively Correlates with Cell Senescence-Related Signaling Pathways in Human PDAC Samples

**Figure 1:**
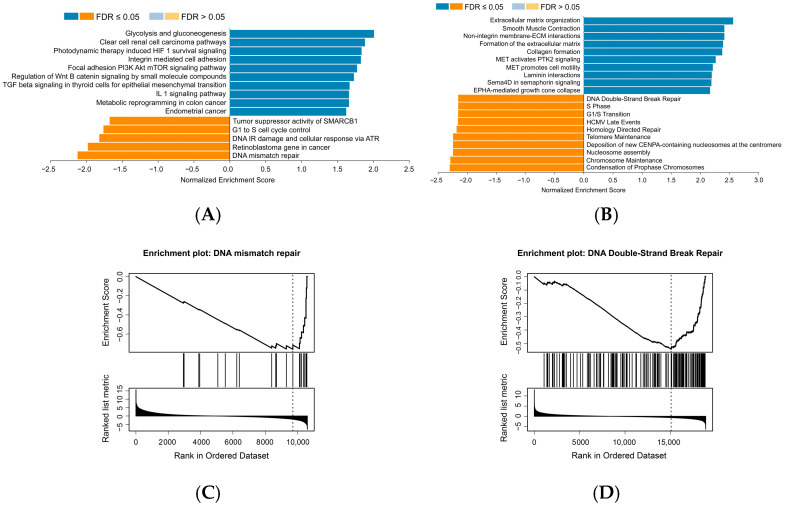
A total of 105 tissue samples from PDAC patients and 44 normal tissue samples from CPTAC were retrieved for an MSLN-related gene set enrichment analysis (GSEA). (**A**) A WikiPathways cancer analysis revealed ten MSLN-positive-related and five MSLN-negative-related categories. (**B**) A Reactome pathway analysis revealed ten MSLN-positive-related and ten MSLN-negative-related categories. (**C**,**D**) Two representatives of DNA damage/DNA repair pathway enrichment plot show that the running scores of these signaling pathways are <0, indicating that MSLN may participate in those biological processes by inversely regulating corresponding pathways.

The CPTAC is a national effort to accelerate the understanding of the molecular basis of cancer through the application of large-scale proteome and genome analyses or proteogenomics [28]. A total of 105 tissue samples from PDAC patients and 44 normal tissue samples from the CPTAC were retrieved to conduct a GSEA in this study. As a result of the WikiPathways cancer analysis (FDR < 0.05 and 1000 permutations), 10 positive related categories and 5 negative related categories were identified as enriched categories, as shown in Figure 1A. The GSEA revealed significant enrichment of MSLN trans-association genes in the pathways of tumor suppressor activity, cell cycle control, DNA damage response, and mismatch repair; the rank plot demonstrated a skewed distribution of these genes toward the bottom of the ranked list, suggesting that MSLN may participate in those biological processes by negatively regulating corresponding pathways. In addition, based on the Reactome pathway analysis (FDR < 0.05 and 1000 permutations), 33 positive related categories and 203 negative related categories were identified as enriched categories, and 10 of the most significant categories and representatives in the reduced sets are shown in Figure 1B. Similarly, the cell cycle-related pathway and DNA repair pathway are significantly enriched in the top MSLN-negative correlation categories. Reactome and WikiPathways are two of the most popular freely available databases for biological pathways [29,30]. Interestingly, both database analyses showed that component genes mainly enriched in cell cycle pathways and DNA damage/DNA repair-related pathways with negative normalized enrichment score (NES) values were negatively correlated with MSLN expression. This suggests that high MSLN expression in PDAC may be associated with the repression or reduced activity of these biological pathways (Figure 1A–D), which is consistent with other reports showing that the senescence pathway is being triggered by a variety of stimuli, including DNA damage, mitochondrial dysfunction, and tumor suppressor loss [31,32,33]. Therefore, we hypothesized that inhibiting the high expression of MSLN in PDAC may induce significant DNA damage/DNA repair activities and thereby activate the senescence pathway.

### 3.2. MSLN Loss Leads to Morphological Changes and Cell Senescence in PDAC Cells

**Figure 2:**
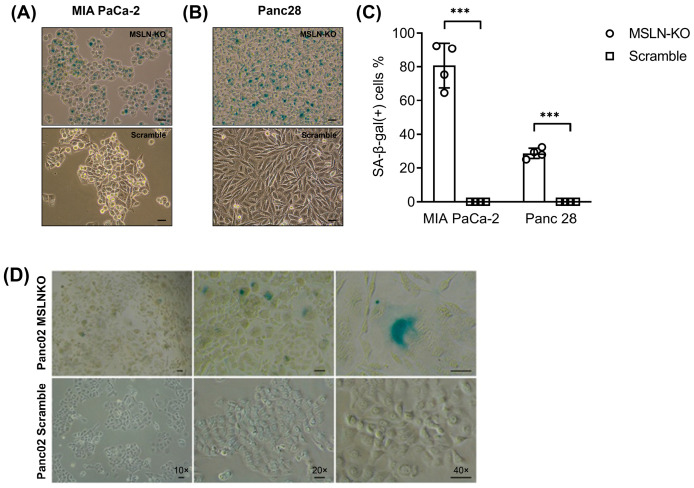
Cell senescence was induced with MSLN loss in PDAC cells. Senescence-associated β-galactosidase (SA-β-gal) staining was performed in MSLN-KO or scramble MIA PaCa2 (**A**) and Panc28 cells (**B**) and observed with 10× objectives, respectively. The proportion of senescence cells in A and B was quantified (**C**). (**D**) SA-β-gal staining was performed in MSLN-KO or scramble Panc02 cells. The Panc02 MSLN-KO and scramble PDAC cells were observed with 10×, 20×, and 40× objectives, respectively. *** *p* < 0.001; unpaired two-tailed *t*-test.

To determine whether MSLN is associated with anti-senescence, we genetically modified MSLN expression levels in both mouse and human PDAC cell lines and determined the changes in these cells when compared with the control cells. Senescence-associated beta-galactosidase (SA-β-gal) is a hydrolase enzyme only found in senescent cells that can catalyze the hydrolysis of β-galactosides into monosaccharides [34]. SA-β-gal activity was strongly associated with senescent cells since it was not detectable in quiescent cells or terminally differentiated cells; therefore, it is now a widely used biomarker in studies of cellular senescence in vitro and in vivo [35]. Here, an SA-β-gal activity assay was performed to distinguish senescent cells from non-senescent cells. We utilized CRISPR/Cas9 technology to target the DNA site (5′-CAACGGCTCGACCCCTGTTG-3′) of the *MSLN* gene to deplete its expression in the PDAC cell lines MIA PaCa-2 and Panc28, and the *MSLN* knockout (MSLN-KO) was subsequently confirmed using PCR-based DNA sequencing (Supplementary Figure S1). SA-β-gal staining showed that the proportion of SA-β-gal-positive cells increases from 0% in the cultures of CRISPR/Cas9 scramble cells to 81% in MIA PaCa-2 MSLN-KO cells (Figure 2A) and to 29% in Panc 28 MSLN-KO cells (Figure 2B). Figure 2C shows the quantification of SA-β-gal-positive cells in MSLN-KO cells vs. scramble control cells. Similarly, we found that MSLN-KO in mouse Panc02 cells also show a significant cellular senescence phenotype (with more SA-β-gal staining, cells become flattened and larger) when compared with the control cells (Figure 2D).

### 3.3. MSLN Is Negatively Correlated with Senescence Regulators

**Figure 3:**
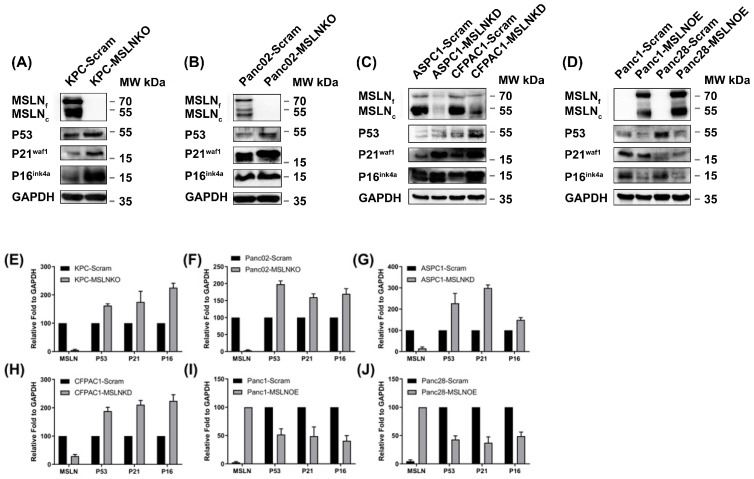
MSLN expressions are negatively correlated with senescence regulators. MSLN KO resulted in elevated levels of senescence markers P16, P21, and P53 in two mouse cell lines: KPC cells (**A**) and Panc02 cells (**B**). MSLN KD resulted in elevated levels of senescence markers P16, P21, and P53 in two human cell lines: ASPC1 and CFPAC1 cells (**C**). MSLN OE resulted in reduced levels of senescence markers P16, P21, and P53 in two human cell lines: Panc1 and Panc28 cells (**D**). (**E**–**J**) The expression levels of MSLN, P53, P21, and P16 in different manipulated mouse and human cells shown above were quantified by using ImageJ software and are presented. Quantification was carried out using triplicate scans and normalized onto GAPDH, and the results are presented in the bar graphs. The original Western blot figures can be found in Supplementary File S1.

P53, p21^waf1^, and p16^INK4a^ are major cell cycle regulators that control cellular senescence [36,37]. Therefore, we further studied the expression levels of these three cellular senescence regulators in PDAC cells before and after *MSLN* genetic modification (knockout, knockdown, or overexpression). As shown in Figure 3, we found that compared with scramble control cells, MSLN-KO or MSLN-KD cells showed elevated expressions of P53, p21^waf1^, and p16^INK4a^, whereas MSLN-OE PDAC cells showed decreased senescence regulator expression (Figure 3A,B: two PDAC mouse cell lines; Figure 3C,D: two PDAC human cell lines). For the mouse PDAC cells, we observed higher fundamental MSLN expression in KPC-Scram than in Panc02-Scram cells; the senescence regulators P53, p21^waf1^, and p16^INK4a^ were dramatically upregulated after MSLN knockout (Figure 3A,B). Similarly, in the human PDAC cell lines, higher expressions of senescence regulators P53, p21^waf1^, and p16^INK4a^ were detected with MSLN knockdown (Figure 3C). The overexpression of MSLN in Panc1 and Panc28 PDAC cells showed reduced expressions of P53, p21^waf1^, and p16^INK4a^ proteins (Figure 3D). The expression levels of MSLN, P53, P21, and P16 in different cells shown in the Western blot were quantified by using ImageJ software (Figure 3E–J). Our findings indicate that MSLN may suppress cellular senescence activation by inhibiting the expression of major cell cycle regulators in PDAC cells.

### 3.4. MSLN Deficiency Induces DNA Damage and Reduces Cell Viability in PDAC Cells

**Figure 4:**
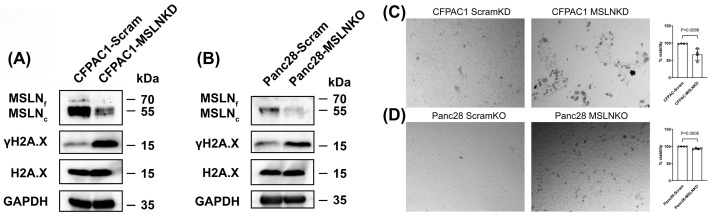
Mesothelin deficiency induced γH2A.X expression indicative of DNA damage and resulted in a moderate but significant reduction in cell viability in PDAC cells. (**A**) MSLN knockdown in CFPAC1 cells resulted in increased expression of DNA damage response protein γH2A.X. (**B**) MSLN knockout in Panc28 cells resulted in increased expression of DNA damage response protein γH2A.X. Antibodies are anti-MSLN (1:1000 dilution), anti-H2A.X (1:1000 dilution), anti-γH2A.X. (1:1000 dilution), and anti-GAPDH (1:1000 dilution). Blots shown are representative of three independent biological replicates. (**C**,**D**) MSLN knockdown or knockout reduces cell viability in PDAC cells, as determined by Trypan Blue exclusion assay. Representative brightfield images of PDAC cells stained with 0.2% Trypan Blue solution. Cell viability was quantified by calculating ratio of number of unstained cells to total number of cells. All photos were taken with 10× objective. Bars represent mean ± SD from three independent biological replicates. ⚪, scramble control cells; □, MSLN-KO or MSLN-KD cells. *p* < 0.05 indicates statistical significance. The original Western blot figures can be found in Supplementary File S1.

To further verify whether the senescence caused by MSLN deficiency involves DNA damage in PDAC cells, we examined the level of γH2A.X, which is phosphorylated H2A.X and a well-established marker of DNA damage that plays a key role in the repair of DNA double-strand breaks. A Western blot analysis revealed a substantial increase in γH2A.X expression in CFPAC1-MSLNKD and Panc28-MSLNKO cells compared to their respective scrambled controls (Figure 4A,B). This increase was not accompanied with changes in the total H2A.X level, indicating that the observed upregulation of γH2A.X occurred due to phosphorylation events rather than changes in overall histone abundance. These findings suggest that the depletion of MSLN activates DNA damage signaling pathways in PDAC cells. We further evaluated the impact of MSLN deficiency on cell viability by using a Trypan Blue exclusion assay. As shown in Figure 4C, CFPAC1 cells with MSLN knockdown (CFPAC1-MSLNKD) exhibited a larger number of non-viable (Trypan Blue-positive) cells compared to the scrambled control (CFPAC1-Scram), indicating increased cell death. A quantitative analysis revealed a significant reduction in cell viability in the MSLNKD group, with viability decreasing to 67% compared to near-complete viability (98.4%) in the control group (*p* = 0.0088). Similarly, Panc28 cells with CRISPR-mediated MSLN knockout (Panc28-MSLNKO) showed a moderate but significant reduction in cell viability relative to Panc28-Scram controls (Figure 4D). Together, these data suggest that the depletion of MSLN compromises cell viability in PDAC cells, potentially through mechanisms associated with increased cellular senescence.

### 3.5. MSLN Suppresses the Production of the Senescence-Associated Secretory Phenotype

**Figure 5:**
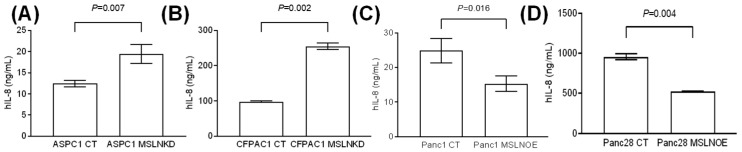
MSLN suppresses SASP production. Senescence-associated secretory phenotype of IL-8 secretion by PDACs was quantified by ELISA. Media from MSLN knockdown/overexpression/control PDAC cell culturing system was collected and applied to ELISA with IL-8 ELISA Kit. (**A**) ASPC1 cells; (**B**) CFPAC1 cells; (**C**) Panc1 cells; (**D**) Panc28 cells. *p* < 0.05 indicates statistical significance based on *t*-test.

Senescent cells have been reported to experience a series of changes before and after, such as morphology change, and the secretion of pro-inflammatory factors termed the senescence-associated secretory phenotype (SASP) [38,39]. SASP-related factors include pro-inflammatory cytokines (such as IL-6 and IL-8) and chemokines (such as CXCL-1/3 and CXCL-10) [40]. Our ELISA data show that MSLN-KD cells showed a significant increase in IL-8 production that was secreted into the cell culture supernatant compared with the control cells (Figure 5A: ASPC1 cells; Figure 5B: CFPAC1 cells), while the MSLN-OE PDAC cells showed obviously downregulated IL-8 levels in the cell culture supernatant (Figure 5C: Panc1 cells; Figure 5D: Panc28 cells). As shown in Figure 5A,B, IL-8 production in ASPC1 or CFPAC1 MSLN-KD cells was significantly greater than in scramble ASPC1 or CFPAC1 cells (19.3 ng/mL vs. 12.4 ng/mL, *p* = 0.007 and 254.7 ng/mL vs. 98.1 ng/mL, *p* = 0.002, respectively). Similarly, in Figure 5C,D, IL-8 production by Panc1 or Panc28 MSLN-OE cells was significantly lower than in Panc1 or Panc28 control cells (15.2 ng/mL vs. 24.7 ng/mL, *p* = 0.016 and 525.0 ng/mL vs. 956.2 ng/mL, *p* = 0.004, respectively).

## 4. Discussion

Our previous studies demonstrated that mesothelin (MSLN) plays a significant role in pancreatic ductal adenocarcinoma (PDAC) by promoting cell proliferation, migration, and invasion while inhibiting apoptosis [12,13,16,17]. Importantly, this study presents novel evidence that MSLN inhibits cellular senescence—a previously underexplored mechanism contributing to its oncogenic role. Our findings align with data from the CPTAC, which show that high MSLN expression in human PDAC is associated with reduced activity in DNA damage response, repair pathways, and cell cycle control mechanisms. This suggests that MSLN helps PDAC cells evade senescence by downregulating these critical pathways, thereby promoting tumor progression. Using CRISPR/Cas9 to knock out MSLN in PDAC cell lines, we found that MSLN-KO cells exhibited hallmark features of cellular senescence, such as growth arrest, morphological changes, and increased senescence-associated β-galactosidase (SA-β-gal) staining. Additionally, we observed elevated levels of senescence-associated molecules (P53, P21^waf1^, and P16^ink4a^) and the pro-inflammatory cytokine IL-8 in MSLN-KO cells. These findings highlight the role of MSLN in preventing cellular senescence in PDAC, suggesting that targeting MSLN may offer a novel approach to enhance the effectiveness of PDAC therapies.

There are limited studies on the role of MSLN in cellular senescence. In our previous study, we examined the effect of MSLN on pancreatic cancer cell proliferation, cell cycle progression, the expression of cell cycle regulatory proteins, and signal transduction pathways in different PDAC cell lines [16,17,18]. Our data show that MSLN-overexpressed PDAC cells enhanced proliferation more than control cells; however, MSLN gene knockdown decreased cell proliferation and postponed the progression of cell entry into the S phase compared with the control cells [13]. Another study indicated that *MSLN*-overexpressed cells upregulated the expressions of cyclin-dependent kinase 2 (CDK2) and cyclin E, which correlated with significantly increased cell proliferation and faster cell cycle progression [12]. The cyclin E/Cdk2 complex is believed to play a key role in the regulation of the cell cycle [41]. Morisaki et al. demonstrated an impaired activation of this complex in senescent cells [42]. Similarly, Yoshida and colleagues showed that CSN5 can control premature senescence by depending on the CDK2/cyclin E axis in human diploid fibroblasts [43]. In addition, CDK2 plays a significant role in suppressing oncogene-induced senescence since CDK2 suppression-induced senescence was detected in various human cancer cell types [44,45]. In another study on ovarian cancer, Li et al. revealed that MSLN displayed the most negative association with several significant KEGG pathways, which were mainly related to the p53 signaling pathway, cellular senescence, and cell cycle [46], but no more data about the details of the MSLN–senescence relationship were provided in their study. The P38 MAPK signaling pathway was reported to be activated in cellular senescence and stress-induced premature senescence triggered by oncogene and culture shock [47,48]; however, there is no evidence that the P38 pathway is significantly altered upon the inhibition of MSLN expression. Therefore, MSLN-induced senescence inhibition may be mainly associated with other signaling responses instead of the P38 MAPK pathway.

**Figure 6:**
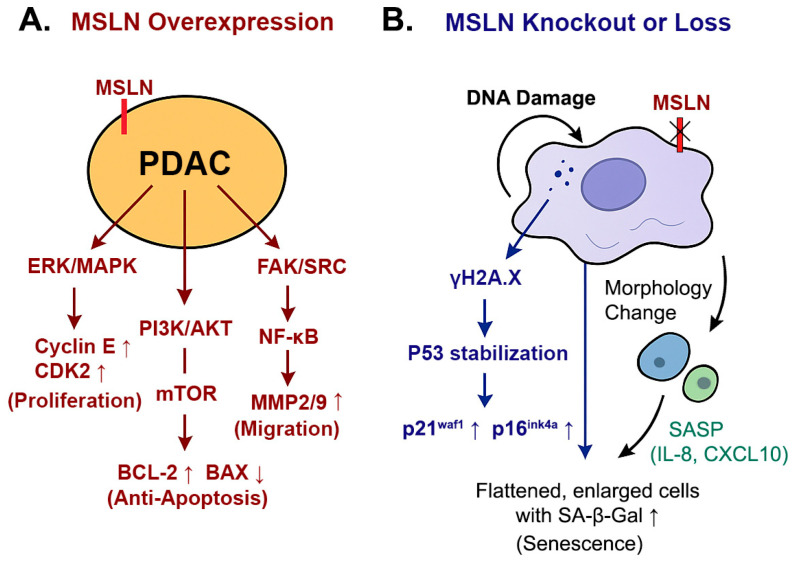
The proposed model of the mesothelin (MSLN)-mediated anti-senescence mechanism (MAAS) in pancreatic ductal adenocarcinoma (PDAC). (**A**) In PDAC cells with high MSLN expression, oncogenic signaling pathways such as PI3K/AKT/mTOR, ERK/MAPK, and FAK/SRC/NF-κB are activated. These promote cell proliferation, survival, and migration while suppressing cellular senescence and apoptosis. (**B**) In MSLN-deficient PDAC cells, the loss of MSLN leads to the accumulation of DNA damage and the activation of the DNA damage response (DDR), as evidenced by increased γH2AX expression. This triggers the upregulation of p53, p21^waf1^, and p16^ink4a^, resulting in cell cycle arrest and the acquisition of senescence phenotypes. Senescent PDAC cells also produce elevated levels of IL-8, a key component of the senescence-associated secretory phenotype (SASP). Collectively, this model illustrates how MSLN suppresses senescence and facilitates tumor progression, representing a novel mechanism termed mesothelin-associated anti-senescence (MAAS).

In the present study, we provided primary evidence that MSLN inhibits senescence in PDAC cell lines. For the first time, our findings revealed that MSLN-associated anti-senescence (MAAS) is accompanied by elevated levels of key senescence regulators, including P53, P21^waf1^, and P16^ink4a^. These proteins play crucial roles in the cellular response to stress, with P53 acting as a transcription factor that can induce cell cycle arrest, apoptosis, or senescence in response to DNA damage [36]. P21, a downstream target of P53, inhibits cyclin-dependent kinases, leading to cell cycle arrest [49]. P16, another important regulator, inhibits CDK4/6, preventing the phosphorylation of RB and thereby enforcing cell cycle arrest [50]. In addition, the fact that MSLN-deficient cells led to a moderate but significant reduction in cell viability further supports the finding that MSLN loss suppresses tumor cell proliferation by inducing a senescent-like state rather than causing overt cytotoxicity. This reduction reflected a shift toward a non-proliferative state consistent with senescence rather than widespread cell death. We also observed increased γH2AX levels in MSLN-deficient cells, indicating the activation of the DNA damage response and supporting the role of MSLN loss in triggering cellular stress and senescence. Together, these findings reinforce the notion that MSLN is critical for maintaining proliferative capacity and genomic stability in pancreatic cancer cells. A schematic model of this MSLN-mediated anti-senescence mechanism is shown in Figure 6.

Our study shows that PDAC senescent cells display an enlarged, flattened cell shape and elevated senescence-associated β-galactosidase activity, a gold standard for identifying senescent cells. Although the component of SASP is very complicated, recent studies demonstrated that numerous unique proteins with immunomodulatory properties are secreted from senescent cells, including inflammatory cytokines, chemokines, growth factors, and matrix metalloproteinases [5]. In the tumor microenvironment, for example, IL-8, IL-12, and tumor necrosis factor (TNF) can recruit immune cells such as M1-like macrophages, enhancing the removal of detrimental senescent and aging cells that are consistent with the tumor suppression effect of senescence [51,52]. To detect the SASP level in cell culture supernatants from senescent PDAC cells induced by MSLN knockdown or overexpression, we performed an IL-8 ELISA. Our data clearly show significantly elevated IL-8 secretion in MSLN knockdown PDAC cell lines compared to the control cells; on the contrary, IL-8 secretion dramatically decreased in MSLN-overexpressed PDAC cells. These findings suggest that the MSLN expression level is inversely correlated with the proportion of PDAC cells in the senescent state, which is consistent with our SA-β-gal data. As another key factor of the SASP, IL-6 can not only promote tumorigenesis and cell proliferation but also exert tumor-suppressive functions depending on the cellular context [5]. Our previous investigation indicated that MSLN-activated NF-kB induces upregulated IL-6 expression, which acts as a growth factor to support PDAC cell proliferation and anti-apoptosis through a novel auto/paracrine IL-6/sIL-6R trans-signaling pathway [17]. Therefore, we hypothesized that the high-MSLN-induced upregulated IL-6 axis (MSLN^H^IL6^H^) is an example of major survival/proliferation signaling in supporting PDAC cell growth; on the contrary, low-MSLN or MSLN-inhibition-induced upregulated IL-6 secretion (MSLN^L^IL6^H^) from senescent cells may play an important role in suppressing tumor progression. More studies are needed to verify this hypothesis.

MSLN knockout studies provide strong mechanistic insights, allowing for these findings to be translated into clinical therapy, which requires a deeper exploration of potential pharmacologic strategies to inhibit MSLN. Currently, several monoclonal antibodies (e.g., amatuximab) and antibody–drug conjugates (e.g., anetumab ravtansine) targeting MSLN are being investigated in preclinical and clinical trials, particularly for mesothelioma [53,54] and ovarian cancer [55,56]. These agents could potentially be repurposed or tested in PDAC settings to evaluate whether pharmacological MSLN inhibition induces tumor senescence and improves therapy response. Furthermore, reported compounds such as synthetic inhibitors have shown promise in modulating mesothelin expression [57], though their mechanisms in the context of senescence remain to be clarified. Another important translational direction involves targeted delivery. Nanoparticle-mediated drug delivery systems or ligand-targeted approaches (e.g., using MUC16-binding peptides) could enhance the selective inhibition of MSLN at the tumor site, minimizing off-target effects [58,59].

Recent progress in senolytic therapies has highlighted their potential in selectively eliminating senescent cells to mitigate age-related diseases and improve cancer treatment outcomes [60,61,62]. Additionally, combination strategies involving senolytics and chemotherapeutic agents have shown promise in reducing therapy-induced senescence and tumor relapse [63,64]. Ongoing clinical trials, including those evaluating dasatinib and quercetin (D + Q) in fibrotic and metabolic diseases, are paving the way for the translational application of senolytics across a range of senescence-associated pathologies, including cancer and degenerative disorders [60,65,66].

This study opens the possibility of combining MSLN-targeted agents with senolytic agents to synergistically induce cancer cell death. Targeting MSLN could therefore be a promising strategy to induce senescence in PDAC cells and improve therapeutic outcomes for PDAC patients. In the future, we will explore a combination strategy using an MSLN blockade in conjunction with senolytics (e.g., dasatinib and quercetin or navitoclax) in PDAC mouse models. While MSLN inhibition is expected to induce a senescent phenotype in PDAC cells via the activation of the P53/P21 and P16/RB pathways, senolytics could be used to selectively clear these senescent tumor cells, potentially reducing the pro-tumorigenic effects of chronic SASP secretion (e.g., IL-6 and IL-8). This sequential treatment strategy may enhance therapeutic efficacy by inducing tumor cell cycle arrest and then eliminating residual senescent cells.

## 5. Conclusions

This study identifies a novel function of MSLN as a key suppressor of cellular senescence in PDAC, expanding its known oncogenic profile beyond proliferation and survival promotion. By modulating MSLN expression in human and murine PDAC models, we demonstrate that MSLN deficiency triggers classical senescence hallmarks—including growth arrest, morphological changes, the activation of the P53/P21^waf1^ and P16^ink4a^ pathways, elevated γH2AX levels, and the increased secretion of SASP factor IL-8—while preserving overall cell viability. These findings define a mesothelin-associated anti-senescence (MAAS) mechanism that enables PDAC cells to evade stress-induced growth arrest and sustain malignant progression. Importantly, our results provide compelling evidence that MSLN acts as a molecular brake on senescence, positioning it as a promising therapeutic target. Disrupting MAAS may not only restore tumor-suppressive senescence programs but also sensitize PDAC cells to senolytic therapies, offering a novel translational strategy to enhance treatment responsiveness and improve clinical outcomes in this aggressive cancer type.

## Data Availability

The CPTAC data we analyzed, which are shown in Figure 1, were derived from the following resource available in the public domain: Proteomic Data Commons (PDC) (https://proteomic.datacommons.cancer.gov/pdc/browse/filters/primary_site:Pancreas, accessed on 6 March 2025).

## Supplementary Materials

The following supporting information can be downloaded at https://www.mdpi.com/article/10.3390/cancers17122058/s1, Figure S1: The characterization of MLSN knocked out PDAC cell lines; File S1: The original Western blot figures.

## References

[1] Wood L.D., Canto M.I., Jaffee E.M., Simeone D.M. (2022). Pancreatic cancer: Pathogenesis, screening, diagnosis, and treatment. Gastroenterology.

[2] Fan M., Deng G., Ma Y., Si H., Wang Z., Dai G. (2024). Survival outcome of different treatment sequences in patients with locally advanced and metastatic pancreatic cancer. BMC Cancer.

[3] Rahib L., Wehner M.R., Matrisian L.M., Nead K.T. (2021). Estimated projection of US cancer incidence and death to 2040. JAMA Netw. Open.

[4] Kumari R., Jat P. (2021). Mechanisms of cellular senescence: Cell cycle arrest and senescence associated secretory phenotype. Front. Cell Dev. Biol..

[5] Dong Z., Luo Y., Yuan Z., Tian Y., Jin T., Xu F. (2024). Cellular senescence and SASP in tumor progression and therapeutic opportunities. Mol. Cancer.

[6] Yamauchi S., Takahashi A. (2025). Cellular senescence: Mechanisms and relevance to cancer and aging. J. Biochem..

[7] Bartkova J., Rezaei N., Liontos M., Karakaidos P., Kletsas D., Issaeva N., Vassiliou L.V.F., Kolettas E., Niforou K., Zoumpourlis V.C. (2006). Oncogene-induced senescence is part of the tumorigenesis barrier imposed by DNA damage checkpoints. Nature.

[8] Marstrand-Daucé L., Lorenzo D., Chassac A., Nicole P., Couvelard A., Haumaitre C. (2023). Acinar-to-ductal metaplasia (ADM): On the road to pancreatic intraepithelial neoplasia (PanIN) and pancreatic cancer. Int. J. Mol. Sci..

[9] Kolodkin-Gal D., Roitman L., Ovadya Y., Azazmeh N., Assouline B., Schlesinger Y., Kalifa R., Horwitz S., Khalatnik Y., Hochner-Ger A. (2022). Senolytic elimination of Cox2-expressing senescent cells inhibits the growth of premalignant pancreatic lesions. Gut.

[10] Yang K., Li X., Xie K. (2023). Senescence program and its reprogramming in pancreatic premalignancy. Cell Death Dis..

[11] Jaber S., Warnier M., Leers C., Vernier M., Goehrig D., Médard J.J., Vindrieux D., Ziegler D.V., Bernard D. (2023). Targeting chemoresistant senescent pancreatic cancer cells improves conventional treatment efficacy. Mol. Biomed..

[12] Bharadwaj U., Li M., Chen C.Y., Yao Q.Z. (2008). Mesothelin-induced pancreatic cancer cell proliferation involves alteration of cyclin E via activation of signal transducer and activator of transcription protein 3. Mol. Cancer Res..

[13] Bharadwaj U., Marin-Muller C., Li M., Chen C.Y., Yao Q.Z. (2011). Mesothelin confers pancreatic cancer cell resistance to TNF-α-induced apoptosis through Akt/PI3K/NF-κB activation and IL-6/Mcl-1 overexpression. Mol. Cancer.

[14] Guo J., Zeng X., Zhu Y., Yang D., Zhao X. (2024). Mesothelin-based CAR-T cells exhibit potent antitumor activity against ovarian cancer. J. Transl. Med..

[15] Rupert P.B., Buerger M., Friend D.J., Strong R.K. (2024). Structural elucidation of the mesothelin-mucin-16/CA125 interaction. Structure.

[16] Li M., Bharadwaj U., Zhang R., Zhang S., Mu H., Fisher W.E., Brunicardi F.C., Chen C.Y., Yao Q.Z. (2008). Mesothelin is a malignant factor and therapeutic vaccine target for pancreatic cancer. Mol. Cancer Ther..

[17] Bharadwaj U., Marin-Muller C., Zhang Y., Li M., Chen C.Y., Yao Q.Z. (2010). Mesothelin overexpression promotes autocrine IL-6/sIL-6R trans-signaling to stimulate pancreatic cancer cell proliferation. J. Surg. Res..

[18] Zhang S., Yong L.K., Li D., Cubas R., Chen C.Y., Yao Q.Z. (2013). Mesothelin virus-like particle immunization controls pancreatic cancer growth through CD8+ T cell induction and reduction in the frequency of CD4+ foxp3+ ICOS^−^regulatory T cells. PLoS ONE.

[19] Nichetti F., Marra A., Corti F., Guidi A., Raimondi A., Prinzi N., de Braud F., Pusceddu S. (2018). The role of mesothelin as a diagnostic and therapeutic target in pancreatic ductal adenocarcinoma: A comprehensive review. Target. Oncol..

[20] Cubas R., Zhang S., Li M., Chen C.Y., Yao Q.Z. (2010). Trop2 expression contributes to tumor pathogenesis by activating the ERK MAPK pathway. Mol. Cancer.

[21] Cao L., Huang C., Zhou D.C., Hu Y., Lih T.M., Savage S.R., Krug K., Clark D.J., Schnaubelt M., Chen L. (2021). Proteogenomic characterization of pancreatic ductal adenocarcinoma. Cell.

[22] Liao Y., Savage S.R., Dou Y., Shi Z., Yi X., Jiang W., Lei J.T., Zhang B. (2023). A proteogenomics data-driven knowledge base of human cancer. Cell Syst..

[23] Elizarraras J.M., Liao Y., Shi Z., Zhu Q., Pico A.R., Zhang B. (2024). WebGestalt 2024: Faster gene set analysis and new support for metabolomics and multi-omics. Nucleic Acids Res..

[24] Fuhrmann-Stroissnigg H., Santiago F.E., Grassi D., Ling Y., Niedernhofer L.J., Robbins P.D. (2019). SA-β-galactosidase-based screening assay for the identification of senotherapeutic drugs. J. Vis. Exp..

[25] Schneider C.A., Rasband W.S., Eliceiri K.W. (2012). NIH Image to ImageJ: 25 years of image analysis. Nat. Methods.

[26] Wang B., Han J., Elisseeff J.H., Demaria M. (2024). The senescence-associated secretory phenotype and its physiological and pathological implications. Nat. Rev. Mol. Cell Biol..

[27] (2020). GraphPad Prism for Windows.

[28] Li Y., Dou Y., Leprevost F.D.V., Geffen Y., Calinawan A.P., Aguet F., Akiyama Y., Anand S., Birger C., Cao S. (2023). Proteogenomic data and resources for pan-cancer analysis. Cancer Cell.

[29] Croft D., Mundo A.F., Haw R., Milacic M., Weiser J., Wu G., Caudy M., Garapati P., Gillespie M., Kamdar M.R. (2014). The Reactome pathway knowledgebase. Nucleic Acids Res..

[30] Kutmon M., Riutta A., Nunes N., Hanspers K., Willighagen E.L., Bohler A., Mélius J., Waagmeester A., Sinha S.R., Miller R. (2016). WikiPathways: Capturing the full diversity of pathway knowledge. Nucleic Acids Res..

[31] Sulli G., Di Micco R., di Fagagna F.D.A. (2012). Crosstalk between chromatin state and DNA damage response in cellular senescence and cancer. Nat. Rev. Cancer.

[32] Yamauchi S., Sugiura Y., Yamaguchi J., Zhou X., Takenaka S., Odawara T., Fukaya S., Fujisawa T., Naguro I., Uchiyama Y. (2024). Mitochondrial fatty acid oxidation drives senescence. Sci. Adv..

[33] Schmitt C.A., Wang B., Demaria M. (2022). Senescence and cancer—role and therapeutic opportunities. Nat. Rev. Clin. Oncol..

[34] Hall B.M., Balan V., Gleiberman A.S., Strom E., Krasnov P., Virtuoso L.P., Rydkina E., Vujcic S., Balan K., Gitlin I. (2016). Aging of mice is associated with p16 (Ink4a)-and β-galactosidase-positive macrophage accumulation that can be induced in young mice by senescent cells. Aging.

[35] Ogrodnik M., Acosta J.C., Adams P.D., di Fagagna F.D.A., Baker D.J., Bishop C.L., Chandra T., Collado M., Gil J., Gorgoulis V. (2024). Guidelines for minimal information on cellular senescence experimentation in vivo. Cell.

[36] Mijit M., Caracciolo V., Melillo A., Amicarelli F., Giordano A. (2020). Role of p53 in the regulation of cellular senescence. Biomolecules.

[37] Takeuchi S., Takahashi A., Motoi N., Yoshimoto S., Tajima T., Yamakoshi K., Hirao A., Yanagi S., Fukami K., Ishikawa Y. (2010). Intrinsic cooperation between p16INK4a and p21Waf1/Cip1 in the onset of cellular senescence and tumor suppression in vivo. Cancer Res..

[38] Cho K.A., Ryu S.J., Oh Y.S., Park J.H., Lee J.W., Kim H.P., Kim K.T., Jang I.S., Park S.C. (2004). Morphological adjustment of senescent cells by modulating caveolin-1 status. J. Biol. Chem..

[39] Herranz N., Gil J. (2018). Mechanisms and functions of cellular senescence. J. Clin. Investig..

[40] Calcinotto A., Kohli J., Zagato E., Pellegrini L., Demaria M., Alimonti A. (2019). Cellular senescence: Aging, cancer, and injury. Physiol. Rev..

[41] Ohtsubo M., Theodoras A.M., Schumacher J., Roberts J.M., Pagano M. (1995). Human cyclin E, a nuclear protein essential for the G1-to-S phase transition. Mol. Cell. Biol..

[42] Morisaki H., Ando A., Nagata Y., Pereira-Smith O., Smith J.R., Ikeda K., Nakanishi M. (1999). Complex mechanisms underlying impaired activation of Cdk4 and Cdk2 in replicative senescence: Roles of p16, p21, and cyclin D1. Exp. Cell Res..

[43] Yoshida A., Yoneda-Kato N., Kato J.Y. (2013). CSN5 specifically interacts with CDK2 and controls senescence in a cytoplasmic cyclin E-mediated manner. Sci. Rep..

[44] Hydbring P., Larsson L.G. (2010). Tipping the balance: Cdk2 enables Myc to suppress senescence. Cancer Res..

[45] Campaner S., Doni M., Hydbring P., Verrecchia A., Bianchi L., Sardella D., Schleker T., Perna D., Tronnersjö S., Murga M. (2010). Cdk2 suppresses cellular senescence induced by the c-myc oncogene. Nat. Cell Biol..

[46] Li Y., Tian W., Zhang H., Zhang Z., Zhao Q., Chang L., Lei N., Zhang W. (2022). MSLN correlates with immune infiltration and chemoresistance as a prognostic biomarker in ovarian cancer. Front. Oncol..

[47] Harada G., Neng Q., Fujiki T., Katakura Y. (2014). Molecular mechanisms for the p38-induced cellular senescence in normal human fibroblast. J. Biochem..

[48] Han X., Lei Q., Xie J., Liu H., Li J., Zhang X., Zhang T., Gou X. (2022). Potential regulators of the senescence-associated secretory phenotype during senescence and aging. J. Gerontol. A.

[49] Al Bitar S., Gali-Muhtasib H. (2019). The role of the cyclin dependent kinase inhibitor p21cip1/waf1 in targeting cancer: Molecular mechanisms and novel therapeutics. Cancers.

[50] Sherr C.J., Beach D., Shapiro G.I. (2016). Targeting CDK4 and CDK6: From discovery to therapy. Cancer Discov..

[51] Wang L., Lankhorst L., Bernards R. (2022). Exploiting senescence for the treatment of cancer. Nat. Rev. Cancer.

[52] Zingoni A., Antonangeli F., Sozzani S., Santoni A., Cippitelli M., Soriani A. (2024). The senescence journey in cancer immunoediting. Mol. Cancer.

[53] Kato T., Tanaka I., Huang H., Okado S., Imamura Y., Nomata Y., Takenaka H., Watanabe H., Kawasumi Y., Nakanishi K. (2025). Molecular mechanisms of tumor progression and novel therapeutic and diagnostic strategies in mesothelioma. Int. J. Mol. Sci..

[54] Kindler H.L., Novello S., Bearz A., Ceresoli G.L., Aerts J.G., Spicer J., Taylor P., Nackaerts K., Greystoke A., Jennens R. (2022). Anetumab ravtansine versus vinorelbine in patients with relapsed, mesothelin-positive malignant pleural mesothelioma (ARCS-M): A randomised, open-label phase 2 trial. Lancet Oncol..

[55] Matsuzawa F., Kamachi H., Mizukami T., Einama T., Kawamata F., Fujii Y., Fukai M., Kobayashi N., Hatanaka Y., Taketomi A. (2021). Mesothelin blockage by Amatuximab suppresses cell invasiveness, enhances gemcitabine sensitivity and regulates cancer cell stemness in mesothelin-positive pancreatic cancer cells. BMC Cancer.

[56] Hassan R., Blumenschein G.R., Moore K.N., Santin A.D., Kindler H.L., Nemunaitis J.J., Seward S.M., Thomas A., Kim S.K., Rajagopalan P. (2020). First-in-human, multicenter, phase I dose-escalation and expansion study of anti-mesothelin antibody–drug conjugate anetumab ravtansine in advanced or metastatic solid tumors. J. Clin. Oncol..

[57] Liu X., Chan A., Tai C.H., Andresson T., Pastan I. (2020). Multiple proteases are involved in mesothelin shedding by cancer cells. Commun. Biol..

[58] Gubbels J.A., Belisle J., Onda M., Rancourt C., Migneault M., Ho M., Bera T.K., Connor J., Sathyanarayana B.K., Lee B. (2006). Mesothelin-MUC16 binding is a high affinity, N-glycan dependent interaction that facilitates peritoneal metastasis of ovarian tumors. Mol. Cancer.

[59] Kaneko O., Gong L., Zhang J., Hansen J.K., Hassan R., Lee B., Ho M. (2009). A binding domain on mesothelin for CA125/MUC16. J. Biol. Chem..

[60] Kirkland J.L., Tchkonia T. (2020). Senolytic drugs: From discovery to translation. J. Intern. Med..

[61] Gasek N.S., Kuchel G.A., Kirkland J.L., Xu M. (2021). Strategies for targeting senescent cells in human disease. Nat. Aging.

[62] Childs B.G., Durik M., Baker D.J., Van Deursen J.M. (2015). Cellular senescence in aging and age-related disease: From mechanisms to therapy. Nat. Med..

[63] Demaria M., O’Leary M.N., Chang J., Shao L., Liu S.U., Alimirah F., Koenig K., Le C., Mitin N., Deal A.M. (2017). Cellular senescence promotes adverse effects of chemotherapy and cancer relapse. Cancer Discov..

[64] González-Gualda E., Pàez-Ribes M., Lozano-Torres B., Macias D., Wilson J.R., González-López C., Ou H.L., Mirón-Barroso S., Zhang Z., Lérida-Viso A. (2020). Galacto-conjugation of Navitoclax as an efficient strategy to increase senolytic specificity and reduce platelet toxicity. Aging Cell.

[65] Justice J.N., Nambiar A.M., Tchkonia T., LeBrasseur N.K., Pascual R., Hashmi S.K., Prata L., Masternak M.M., Kritchevsky S.B., Musi N. (2019). Senolytics in idiopathic pulmonary fibrosis: Results from a first-in-human, open-label, pilot study. eBioMedicine.

[66] Hickson L.J., Prata L.G.L., Bobart S.A., Evans T.K., Giorgadze N., Hashmi S.K., Herrmann S.M., Jensen M.D., Jia Q., Jordan K.L. (2019). Senolytics decrease senescent cells in humans: Preliminary report from a clinical trial of Dasatinib plus Quercetin in individuals with diabetic kidney disease. eBioMedicine.

