# Supplementary material for: Mesothelin-Associated Anti-Senescence Through P53 in Pancreatic Ductal Adenocarcinoma
Source: Cancers (Basel). 2025 Jun 19;17(12):2058. doi: 10.3390/cancers17122058 (PMC12191213; doi:10.3390/cancers17122058)
Supplement: Supplementary file 1 [file cancers-17-02058-s001.zip › cancers-3580614-supplementary.pdf]

Supplementary Materials

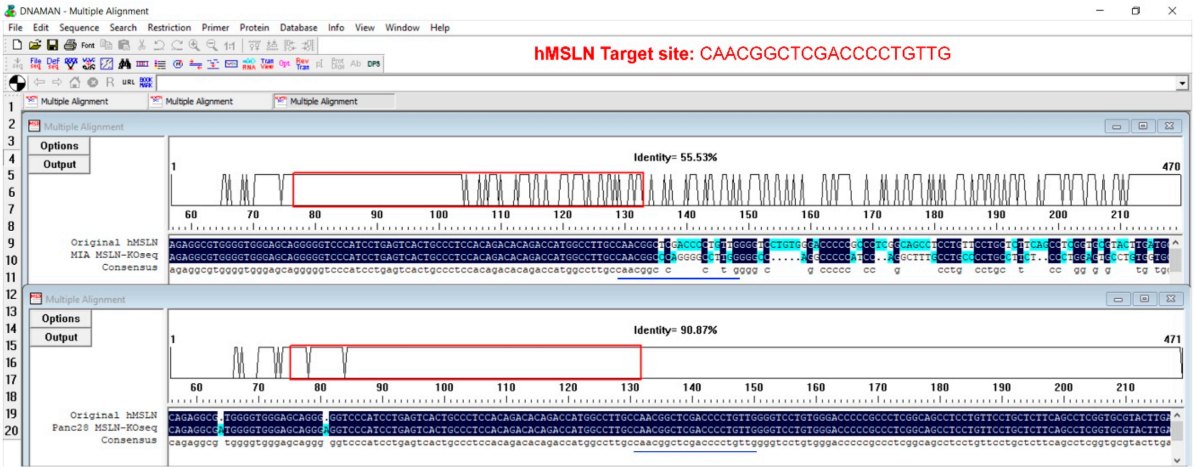

Figure S1. The characterization of MLSN knocked out PDAC cell lines. The red boxes displayed regions of alignment between the original MSLN sequence and MLSN sequencing data from MIA Paca2 MSLN-KO or Panc28 MSLN-KO cells respectively. The blue lines underlined the CRISPR/CAS9-targeted MSLN site sequence CAACGGCTCGACCCCTGTTG. There are many missense mutations at the target site according to the sequencing data of MIA Paca2 MSLN-KO cells. There are two insertional mutations upstream of the target site of Panc28 MSLN-KO cells, which cause the frameshift mutation.

Supplementary File S1. Original Blots

Figure 3A

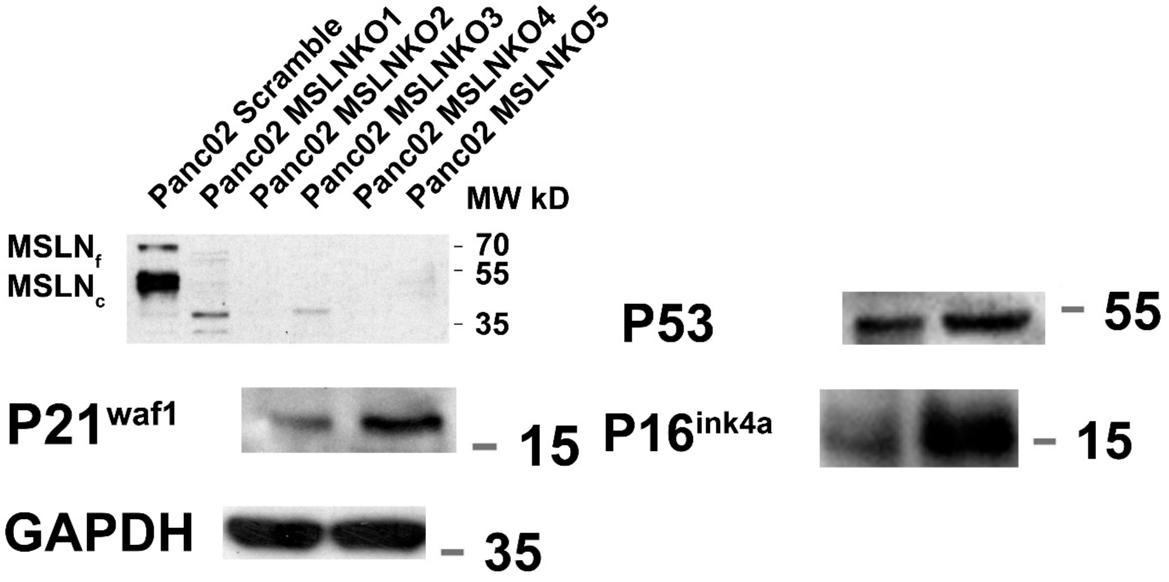

Figure 3B

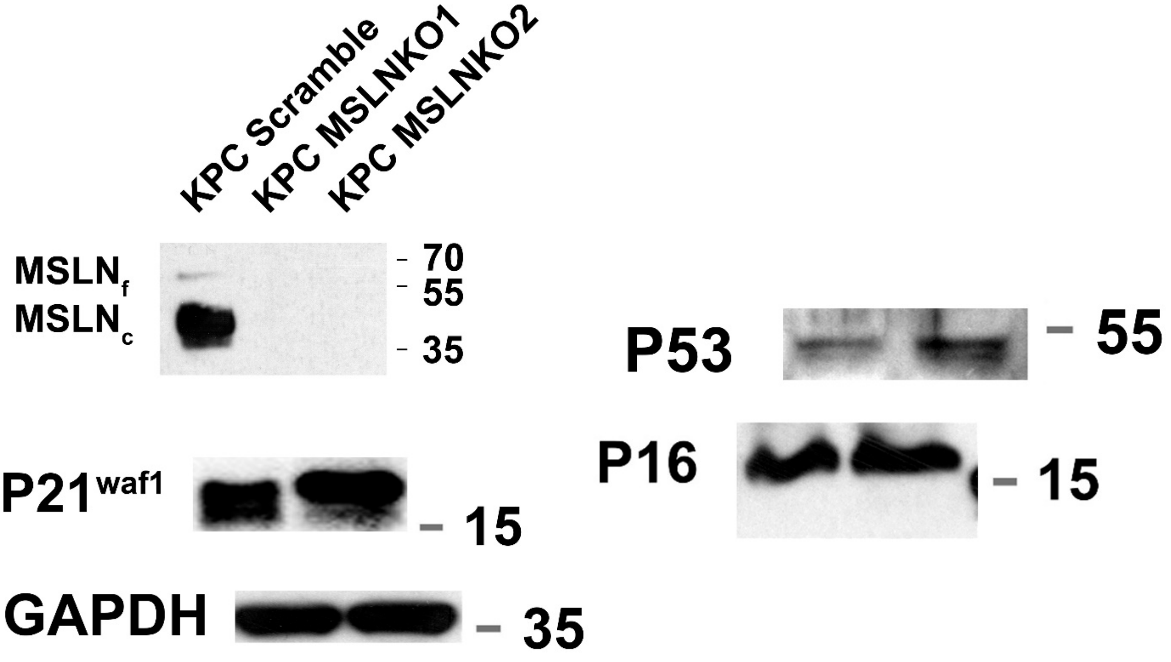

Figure 3C

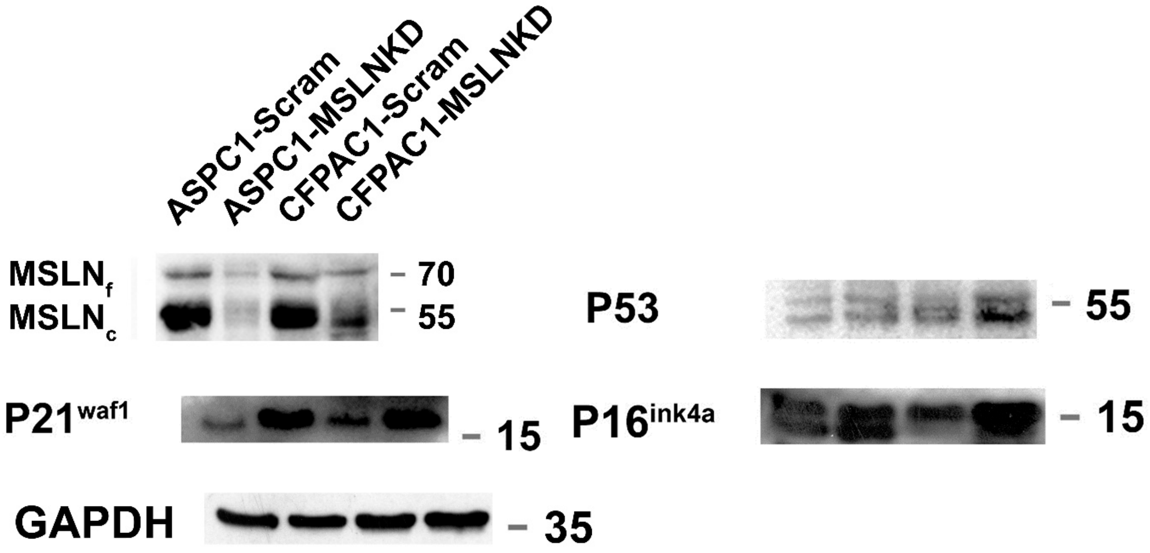

Figure 3D

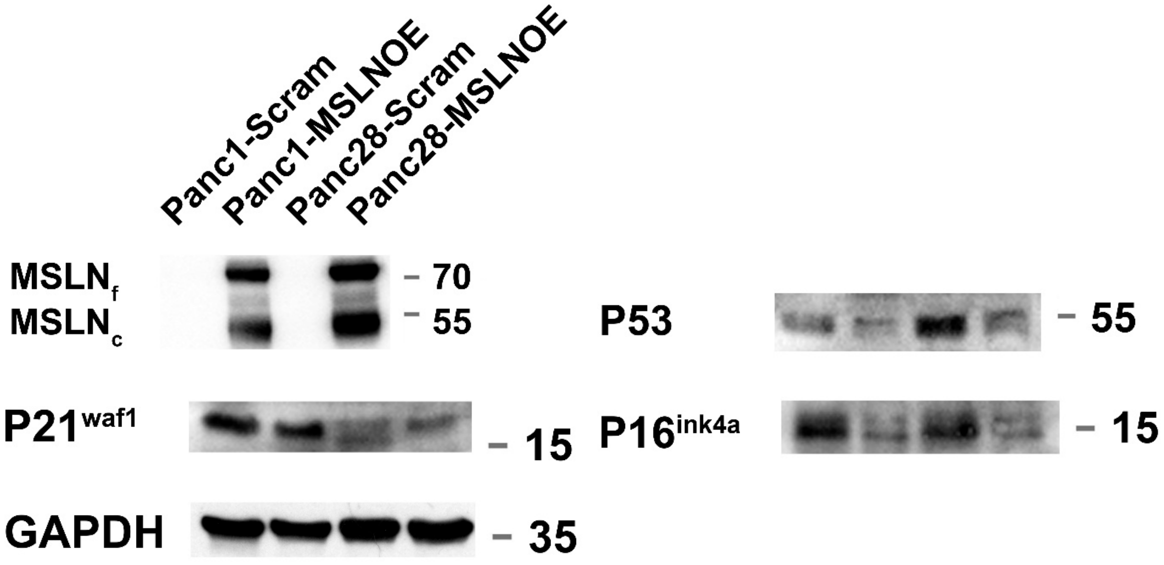

Figure 4A

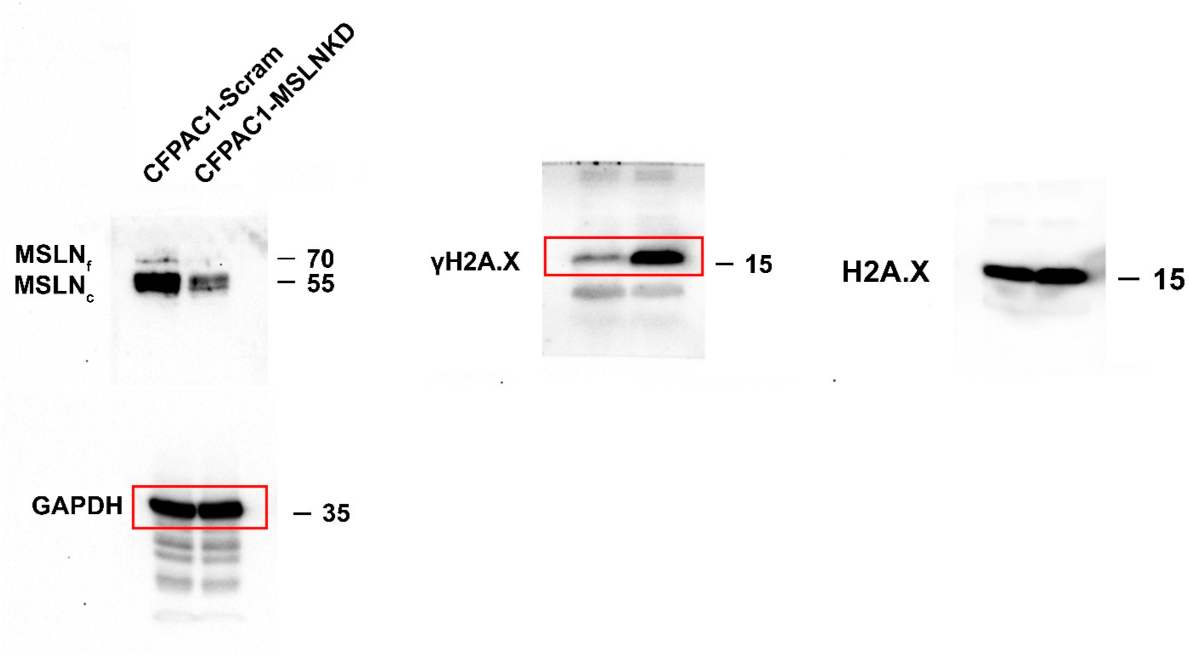

Figure 4B

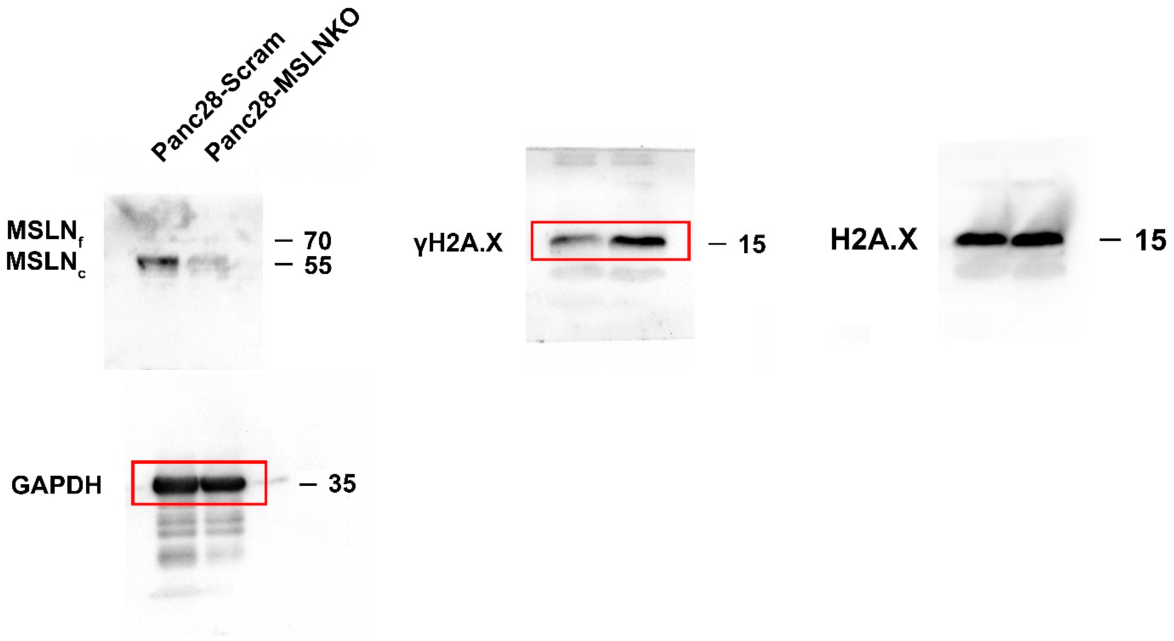

Panc28-Scram
Panc28-MSLNKO
MSLN$_f$
MSLN$_c$
— 70
— 55
γH2A.X
— 15
H2A.X
— 15
GAPDH
— 35
